# Efficient determination of the accessible conformation space of multi-domain complexes based on EPR PELDOR data

**DOI:** 10.1007/s10858-023-00426-3

**Published:** 2023-11-15

**Authors:** Sina Kazemi, Anna Lopata, Andreas Kniss, Lukas Pluska, Peter Güntert, Thomas Sommer, Thomas F. Prisner, Alberto Collauto, Volker Dötsch

**Affiliations:** 1 Institute of Biophysical Chemistry and Center for Biomolecular Magnetic Resonance, Goethe University, Max-von-Laue Str. 9, 60438 Frankfurt am Main, Germany; 2 Signals GmbH & Co. KG, Altenhöferallee 3, 60438 Frankfurt am Main, Germany; 3 Max-Delbrück Center for Molecular Medicine, Robert-Rössle-Str. 10, 13125 Berlin-Buch, Germany; 4 Institute of Molecular Physical Science, ETH Zurich, Vladimir-Prelog-Weg 2, 8093 Zürich, Switzerland; 5 Institute for Biology, Humboldt Universität zu Berlin, Invalidenstrasse 43, 10115 Berlin, Germany; 6 Institute of Physical and Theoretical Chemistry and Center for Biomolecular Magnetic Resonance, Goethe University, Max-von-Laue Str. 9, 60438 Frankfurt am Main, Germany; 7 Present Address: Department of Chemistry, Imperial College London, Molecular Sciences Research Hub, London, W12 0BZ UK; 8 Present Address: Department of Molecular Immunology and Toxicology and the National Tumor Biology Laboratory, National Institute of Oncology, Budapest, Hungary

**Keywords:** PELDOR/DEER, Conformation space, Structural modelling, K48-linked ubiquitin chains, E2 enzymes

## Abstract

Many proteins can adopt multiple conformations which are important for their function. This is also true for proteins and domains that are covalently linked to each other. One important example is ubiquitin, which can form chains of different conformations depending on which of its lysine side chains is used to form an isopeptide bond with the C-terminus of another ubiquitin molecule. Similarly, ubiquitin gets covalently attached to active-site residues of E2 ubiquitin-conjugating enzymes. Due to weak interactions between ubiquitin and its interaction partners, these covalent complexes adopt multiple conformations. Understanding the function of these complexes requires the characterization of the entire accessible conformation space and its modulation by interaction partners. Long-range (1.8–10 nm) distance restraints obtained by EPR spectroscopy in the form of probability distributions are ideally suited for this task as not only the mean distance but also information about the conformation dynamics is encoded in the experimental data. Here we describe a computational method that we have developed based on well-established structure determination software using NMR restraints to calculate the accessible conformation space using PELDOR/DEER data.

**Supplementary Information:**

The online version contains supplementary material available at 10.1007/s10858-023-00426-3.

## Introduction

Structure determination by Nuclear Magnetic Resonance (NMR) spectroscopy is a well-established method. The main structural information is obtained from NOE measurements that are translated into distance restraints between hydrogen atoms. Typically, this includes setting an upper distance limit derived from the volume or the signal height of NOE peaks in multi-dimensional NMR spectra and a lower distance limit defined by the sum of the van der Waals radii of the hydrogens. Instead of a precise distance, a distance range is thus created that reflects the dependence of the NOE on dynamics and flexibility, which cannot be determined precisely. In addition, the number of distance restraints—supplemented with dihedral angle restraints obtained from chemical shift data or coupling constants—is typically significantly lower than the number of degrees of freedom. Structure calculation software packages such as CYANA (Guntert et al. 1997; Guntert and Buchner 2015) therefore use a molecular dynamics-derived algorithm with strongly simplified physical potentials to sample the conformation space restricted by the NMR restraints. This procedure results in structural bundles instead of a single structure. The tighter the bundle, the better defined is the structure. A lack of restraints can, however, also be due to internal flexibility and is typically seen in loop regions that show multiple conformations. In structures determined by X-ray crystallography, such regions are often not visible because of low electron density. NMR spectroscopy is well suited for characterizing the conformation space of such regions. However, the NOE effect can only characterize the local conformation space due to the short range of the nuclear dipolar interaction. To include also information about longer distances, NOE restraints are often combined with paramagnetic relaxation enhancement (PRE) (Battiste and Wagner 2000; Reckel et al. 2011; Pruneda et al. 2011; Longinetti et al. 2006) data, fluorescence resonance energy measurements or small angle X-ray scattering data (Pruneda et al. 2011). In contrast to the small magnetic moment of the nuclei, the larger magnetic moment of the electron enables the measurement of dipolar interactions between unpaired electrons over much larger distances using electron paramagnetic resonance (EPR) spectroscopy. In PELDOR (also known as DEER) experiments (Milov et al. 1984; Pannier et al. 2000), the dipolar interaction between two unpaired electrons can be measured over long-range distances (1.8–10 nm); the resulting time-domain traces can be converted through parameter-free processing into distance probability distributions (Jeschke et al. 2002), which, besides reporting on the mean distance between the paramagnetic centers, encode in their width and shape information about the conformation dynamics. The recently developed 5-pulse DEER (Borbat et al. 2013) and 7-pulse Carr-Purcell (CP) PELDOR sequences (Spindler et al. 2015) additionally provide an increased accuracy of these distance distributions in the case of long distances with broad distributions.

EPR PELDOR measurements are therefore ideally suited for investigating the conformation space when large, global movements are involved, as for example seen in the transport mechanism of membrane-bound chloride CLC transporters (Chavan et al. 2020), MATE transporters (Jagessar et al. 2020), ABC transporters (Barth et al. 2018; Dastvan et al. 2019)or the relative orientations of the POTRA domains of the membrane protein Omp85 from cyanobacteria (Dastvan et al. 2016). An additional class of systems that can be studied by EPR PELDOR are systems of covalently linked but weakly interacting protein modules. Examples are polyketide and non-ribosomal peptide synthetases in which many domains are expressed as a poly-domain protein with flexible linkers that interact with each other to allow the transfer of the product of one reaction center as the substrate to the following reaction center (Strieker et al. 2010; Koglin et al. 2008). Other examples include the interaction of ubiquitin moieties or ubiquitin-like proteins within ubiquitin chains or within covalent E2 and E3 enzyme-ubiquitin complexes (Pohl and Dikic 2019; Komander and Rape 2012). These interactions are often relatively weak (in the tens of micromolar range) but have a strong influence on the cellular response triggered by these interactions (von Delbruck et al. 2016; Dikic et al. 2009). For example, ubiquitin chains linked via an isopeptide bond between the C-terminus of one moiety and the lysine side chain of K48 typically result in the proteasomal degradation of the tagged protein (Kwon and Ciechanover 2017; , Thrower et al. 2000). In contrast, a connectivity via the side chain of K63 is an important signal for DNA repair and other cellular processes (Komander and Rape 2012). In order to understand the signal code contained within these chains and to get mechanistic insight into weak protein-protein interactions that cannot be described as a single complex, the characterization of the entire conformation space spanned by these molecules and its modulation by interaction partners is a prerequisite. We applied EPR PELDOR spectroscopy to characterize such interactions. To visualize the conformation space of these systems we have adapted calculation techniques from NMR-based structure determination.

## Results

### Determination of the conformation space of weakly interacting but covalently bound systems

So far we have studied two distinct macromolecular systems that are characterized by weak interactions using EPR PELDOR spectroscopy. In a previous study, we investigated a di-ubiquitin chain in which the two moieties are linked via a covalent bond between the side chain of K48 of the acceptor molecule and the C-terminus of the donor molecule (Kniss et al. 2018). In the currently investigated system, the C-terminus of ubiquitin was covalently linked to the side chain of K89 of the E2 enzyme Ubc7 linked to the U7BR peptide from yeast (von Delbruck et al. 2016). The U7BR peptide is a domain of the Cue1 protein and binds to the backside of the Ubc7 E2 ligase and stabilizes it (Metzger et al. 2013). In cells, this interaction is also required to recruit the E2 enzyme to the membrane of the endoplasmic reticulum (Biederer et al. 1997). We have used throughout our investigations a single chain construct in which the U7BR peptide is linked via a glycine-serine linker to the C-terminus of Ubc7. K89 is a mutant of the wild type C89 amino acid of Ubc7 and was chosen to produce a more stable bond between Ubc7-U7BR and ubiquitin (Plechanovova et al. 2012) (as the lysine side chain is longer than the cysteine side chain, this stabilized bond, however, might lead to an increased flexibility). Both systems—ubiquitin chains as well as E2-ubiquitin conjugates—play important roles in cellular signal transduction and characterizing these complexes is important to understand their biological functions at the molecular level. The binding constants of ubiquitin with its numerous interaction partners (e.g. other ubiquitin molecules, E2 and E3 enzymes as well as a myriad of different ubiquitin binding domains) have been determined and are very often in the range of − 10 µM (Dikic et al. 2009). The combination of such weak interactions with a covalent attachment results in a conformation space populated by many different states with different probabilities. In case of the interaction of E2 enzymes with conjugated ubiquitin, a combination of NMR chemical shift differences, PRE measurements and SAXS data had been used to show that the UbcH5c–ubiquitin system shows a wide distribution of relative orientations of both proteins with respect to each other, while this distribution for the Ubc13–ubiquitin conjugate was far more restricted with ubiquitin preferentially interacting via its Ile44 centered hydrophobic patch with helix 2 of the E2 enzyme (Pruneda et al. 2011).

### Previous protocol for calculating the conformation space of di-ubiquitin

In our previous investigation of the conformation space of di-ubiquitin we had used an approach based on attaching MTSL spin label via a cysteine side chain to different positions within both ubiquitin molecules of a di-ubiquitin chain (Kniss et al. 2018). Subsequently, EPR PELDOR measurements were performed and analyzed using the Gaussian model-based approach implemented in the DeerAnalysis2016 (Jeschke et al. 2006) or DD (Stein et al. 2015) software packages, which yields the distance distribution between the two spin labels. The results of the analysis were cross-checked against the distributions obtained using the Tikhonov-regularized model-free approach implemented in DeerAnalysis2016, always showing almost identical results. For the purpose of the subsequent determination of the conformation space, the model-based approach was preferred because of the absence of additional components in the distance probability distributions arising, for instance, from the specific choice of the regularization parameter or from the intermolecular component by which the signal has to be divided prior to the analysis. Besides, the Tikhonov regularization approach may be unsuitable for distance probability distributions characterized by the coexistence of narrow and broad components, as they would result for example from the equilibrium between an open and a closed conformation.

To determine and visualize the populated conformation space based on the measurement of several of these distance distributions, we developed protocols for the software package CYANA (combined assignment and dynamics algorithm for NMR applications) (Guntert et al. 1997; Guntert and Buchner 2015) that is used to determine structures of biological macromolecules based on NMR-derived restraints. For these calculations, a CYANA library entry for the side chain of MTSL-labelled cysteine was introduced (Kniss et al. 2018). In our original implementation of PELDOR-based ensemble distribution calculations that we used for the determination of the conformation space of di-ubiquitin, we created in the first step a conformation ensemble with a broad distance distribution between both ubiquitin molecules using CYANA version 3.9. A virtual atom was placed in the center of the terminal N–O bond of each spin-labelled amino acid side chain as reference point for distance restraints. The dihedral angles of the spin-labelled sidechain were restricted based on a rotamer library generated for each labelled residue by MMM2015.1 in 298 K mode (Polyhach et al. 2011). These rotamer library restraints were included in our calculations using an extension of CYANA (Guntert et al. 1997; Guntert and Buchner 2015)that takes into account all rotamers from the given rotamer library; this was realized by a new type of restraint that has a value of 0 (no contribution to the target function) if the spin-labelled sidechain adopts a conformation included in the library and increases with increasing deviation from the allowed conformations in this library. Subsequently, from the set of experimentally available EPR distance restraints—five, in this specific case (Kniss et al. 2018)—a conformation ensemble was built by varying systematically and independently each distance between spin-labelled positions within the full range of the corresponding distance probability distribution. For this purpose, the aforementioned ranges, identified by the condition that the probability distribution has to be above a given threshold (in the specific case 0.1% of the maximum value), were divided into 0.5-nm bins and an additional term was added to the CYANA target function to enforce the fulfillment of the specific combination of inter-spin distances; similarly to how NOE-derived restraints are taken into account, this term gives no contribution if each distance is within the given bin and adds a quadratic penalty with increasing deviation outside of the bins (Guntert et al. 1997).

The conformation ensemble generated according to this procedure contained all possible combinations of the five measured distances within each of these distance ranges. Overall, 51,000 structure calculations were performed. For each distance combination, a bundle of the 20 conformers with the lowest target function out of 100 calculated structures was generated. All structures showing van der Waals collisions between the two ubiquitin moieties or not fulfilling the above-described distance restraints were discarded to generate a collisionfree conformation ensemble of − 3.7 × 10^5^ (Pruneda et al. 2011) models. This ensemble represents the accessible conformation space that is consistent with the upper and lower boundaries of the PELDOR measurements.

To interpret and visualize the conformation ensemble of di-ubiquitin, probabilities were assigned to each of the conformers as described below. The results of that study enabled a better understanding of shifts in the conformation ensemble of multimers due to different experimental conditions and the influence of modulating ligands. However, this method was a brute-force approach requiring a complete structure calculation for each distance combination. Thus, while the proposed approach was successful in determining the conformation space of di-ubiquitin based on five PELDOR restraints (Kniss et al. 2018), the exponential increase in computational time with the number of restraints hinders its application to systems where more restraints are measured to achieve higher accuracy. Furthermore, the introduction of tight distance restraints between the spin labels led to a number of disrupted conformers that had to be filtered out after the conformation sampling.

### The more efficient calculation protocol and its application to the Ubc7-U7BR–ubiquitin conjugate

With an increasing number of restraints—as we had planned for the investigation of the Ubc7-U7BR–ubiquitin system, this particular implementation would have resulted in unrealistic long computation times. To allow for a more efficient conformation sampling comparable to applications of CYANA in NMR, it is necessary to introduce the PELDOR-derived distance restraints directly into the CYANA target function. As previously mentioned, NMR distance restraints can be taken into account during the structure calculation with CYANA by introducing them as upper and lower distance limits between two atoms or atom groups; a violation of a distance restraint is represented by a quadratic penalty added to the target function, whereas a restraint does not contribute to the target function if the distance is within the specified upper and lower limits.

 Compared to NMR distance restraints, however, PELDOR-based distance distributions between distant spin labels are rather broad, especially if located on different proteins. Thus, introducing this additional kind of restraint into the CYANA target function as simple distance faces two major obstacles (1). As the CYANA molecular dynamics calculation is performed in torsion angle space, each term in the target function must be a differentiable function of the torsion angles with efficiently computable first partial derivatives (2). The simulated annealing approach implemented in CYANA results predominantly in solutions near or at the minimum of the target function. As a result, conformations that are near the highest populated distance in the PELDOR distance distribution might be over-represented in the resulting structure bundle.

**Fig. 1:**
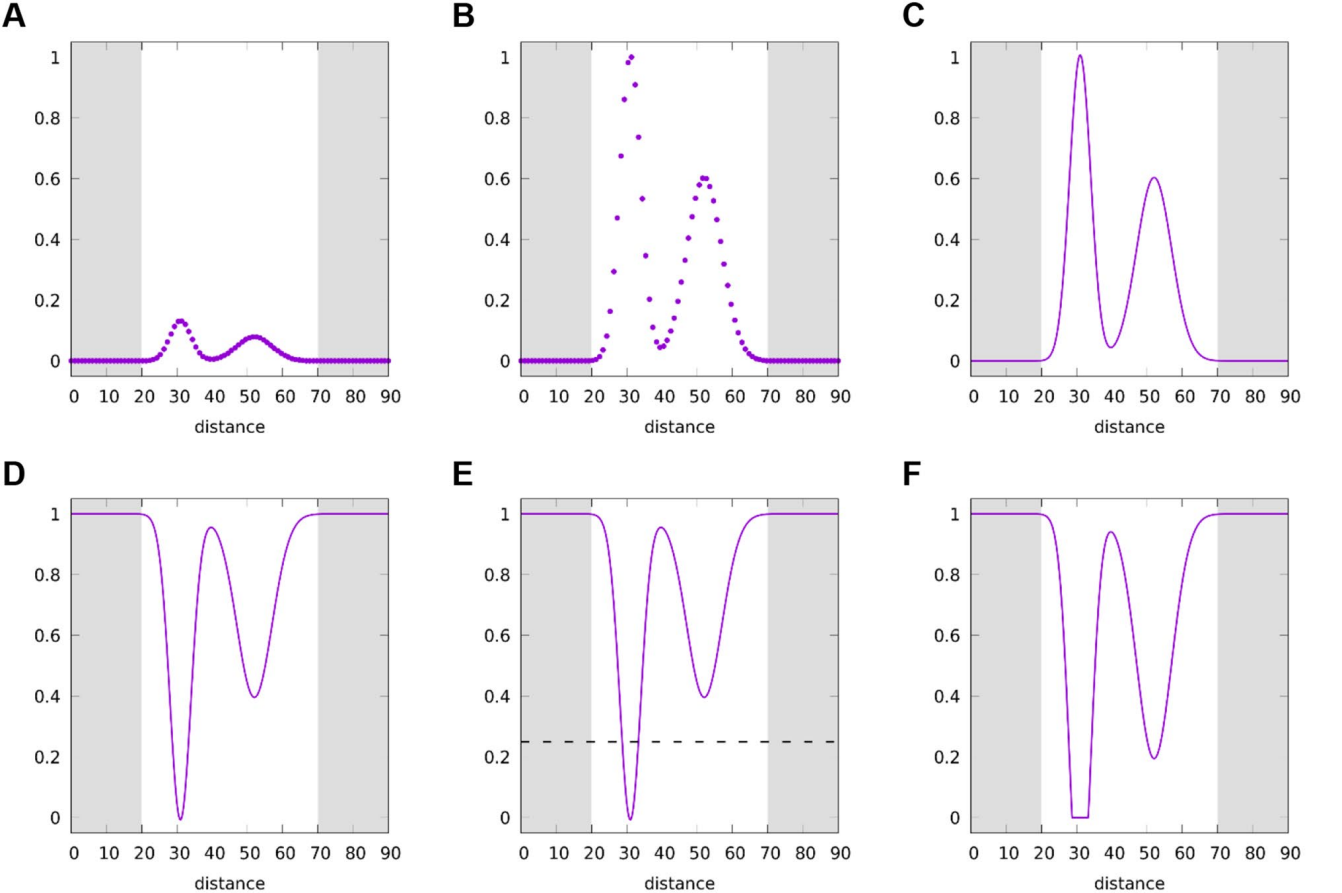
Transforming a discrete experimental distance distribution to a CYANA target function term. In each panel the horizontal axis is the distance expressed in Å between the respective spin labels. Panel **A** shows the discrete probability density function, which is normalized in (**B**), and made continuous using splines in (**C**). To obtain a penalty term for the CYANA target function the distribution is inverted (**D**) such that regions of zero probability density yield the highest penalty value of 1. In addition, low values (below 0.25 – dotted line in E) are truncated, creating a plateau in the rescaled target function (**F**) to lower the strain in the system

To overcome these issues, we devised the following approach. At the first step of the structure optimization, the experimental distance distributions are included into the CYANA target function as additive terms that contribute 0 to the target function if an inter-spin distance is in agreement with the corresponding experimental distance distribution and a value > 0 in relation to the deviation from the distance distribution. To this end, each experimental distance distribution is represented by $N$ discrete pairs $\left\{ {(r_{1} ,~~p_{1} ),~...,(r_{N} ,~~p_{N} )} \right\}$, where ${r}_{i}$is a distance value and ${p}_{i}$ the corresponding probability (Fig. 1A), which is converted into a continuous distance distribution $P\left(r\right)$ (Fig. 1C) using Bézier curves such that $P\left({r}_{j}\right)\approx {p}_{j}$. This last step ensures that analytical derivatives can be formulated, thus addressing the first issue. The resulting target function term $T$ is

$$T\left(r\right)=A \text{m}\text{a}\text{x}\left(0, 1-\frac{P\left(r\right)}{{p}_{\text{m}\text{a}\text{x}}c}\right)$$

where $A$ is a weighting factor, $p_{{\max }} = \max \left\{ {p_{1} ,...,p_{N} } \right\}$, and $c=0.75$ a cutoff threshold (Fig. 1E). This target function term adds a penalty only where the normalized probability $P\left(r\right)/{p}_{\text{m}\text{a}\text{x}}$ is below the cutoff threshold $c$ (Fig. 1F).

As mentioned before, calculations deploying a target function with this kind of term result in structure ensembles near the maximal probability in the distance distribution. In order to allow for a full sampling of the given distance distribution (addressing the above-mentioned obstacle 2), we chose an iterative approach in which the distance distribution of the already calculated ensemble is subtracted from the given experimental distribution. Thus, after the first iteration high-probability regions, that because of this would also be highly sampled, are lowered in probability with each successive iteration. To this aim, a Gaussian function with a given standard deviation $\sigma$ (*vide infra* for the numerical value) and curve area is subtracted from the distance distribution for each distance observed in a calculated conformer. If the number of conformations generated over all iterations is $n$, the area below each of these Gaussians is set to $1/n$. Summing these Gaussians for all conformations results therefore in a calculated distance distribution with the same area as the experimental distance distribution, which is normalized to unit area. Subtracting all the Gaussians leads to a nearly flat distribution towards the last iterations, whereby values below zero are set to 0 to avoid invalid negative values in the distribution.

**Fig. 2:**
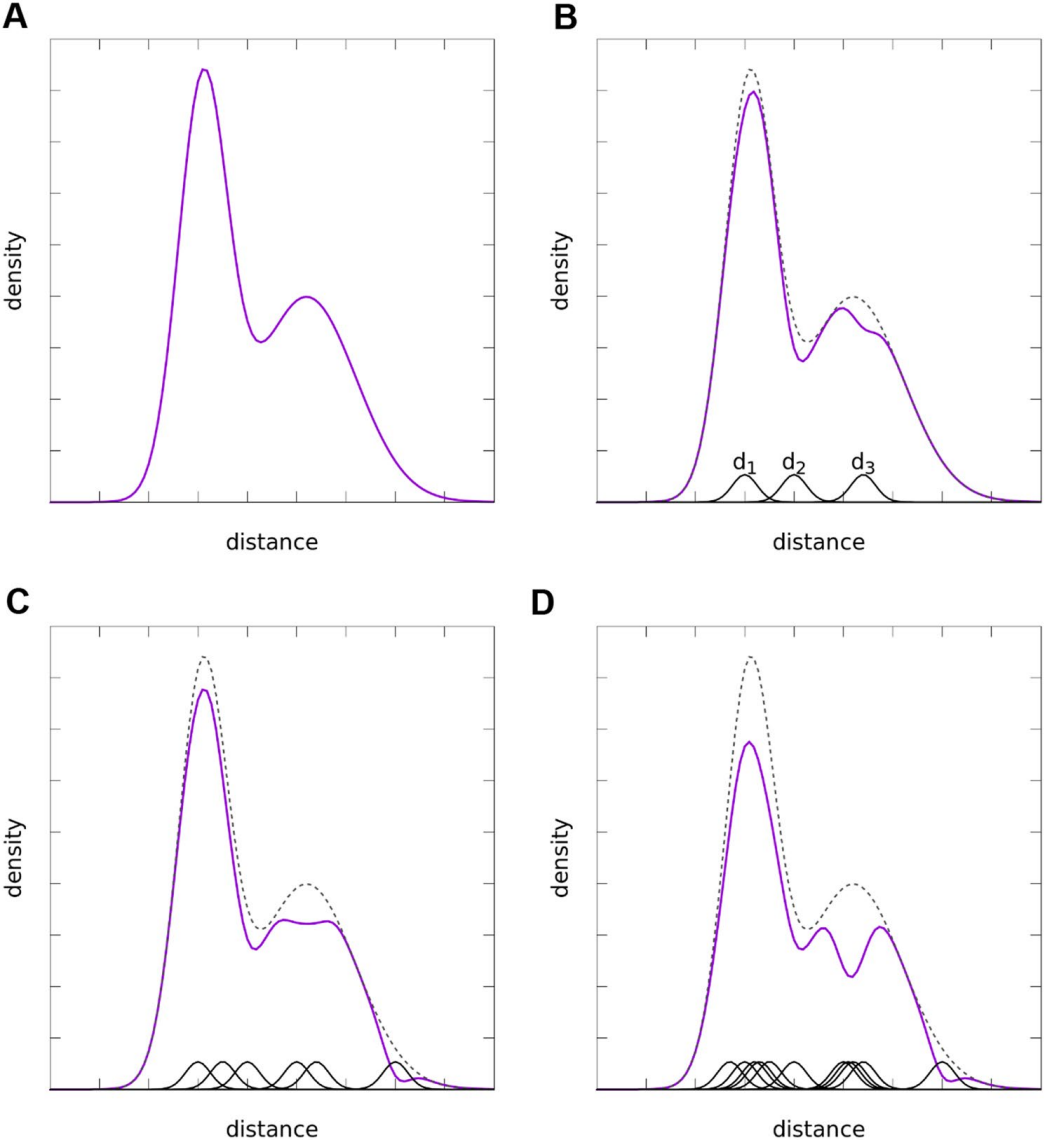
Illustration of the difference between an example starting distance probability distribution derived from EPR measurements (**A**) and the distribution after the generation of structures in the ensemble (magenta). The change in the distance distribution is shown after the generation of 3 (**B**), 6 (**C**), and 11 (**D**) structures. The original experimental distance distribution is shown as a dashed line and the accumulated Gaussians for the distances of each structure as black curves

An example for the resulting distribution after the calculation of three, six and eleven structures is shown in Fig. 2.

This approach was applied in the investigation of the conformation space of ubiquitin covalently attached to Ubc7-U7BR. We determined PELDOR-based distance distributions for a total of 17 spin label pairs (in each case one in the ubiquitin and one in the E2 enzyme; Supplementary Table S1). A cutoff value of *c* = 0.75 and the weighting factor *A*= 10.0 Å^2^ (Guntert and Buchner 2015) were set; owing to its large weight, the additional term overweighs other contributions to the CYANA target function, therefore driving the calculation to fulfill the experimental PELDOR restraints.

Despite the fact that the distance distributions were derived by several experiments with two spin labels per molecule, in the calculation all spin labels were modelled in one system assuming equal dynamical behavior of the molecule in each individual experiment. 250 iterations were performed, whereby in each iteration 100 structures were calculated and 10 structures with the lowest values of the target function were selected (the selection of the top 10% of the calculated conformations is a well-established method in NMR structure calculation to ensure convergence). For the first iteration the area-normalized distance probability distributions obtained from the analysis of the PELDOR data were used, whereas at each successive iteration a correction was applied as described above by subtracting for each double mutant the inter-spin distance distribution derived from the *i*^th^ structure; this was represented by a Gaussian distribution with a standard deviation $\sigma$ of 2.5 Å and an area of 1/2500, thus yielding at the end of the calculation a curve with unit area. In other words, at each point of the calculation and for each distance the sum of the probability distributions of all the already calculated structures is subtracted from the corresponding PELDOR-derived probability distribution.

The result is an ensemble of 2500 structures that resembles the experimental distance probability distributions without violating any of the restraints of the structure calculation. Crystal structures of Ubc7-U7BR (PDB ID: 4JQU) and ubiquitin (PDB ID: 1UBQ) were used as templates for the calculations. The residue missing in the crystal structure 4JQ4 (97–102 of Ubc7 as well as the di-Gly linker connecting Ubc7 to U7BR of Cue1) and the isopeptide bond between G76 of ubiquitin and K89 of Ubc7 were all introduced into the structure from the default CYANA residue templates. The rotatable torsional angles of these residues were set to random values at the beginning of each calculation. In addition to the experimentally derived distance ensemble restraints, the backbone of the following parts of the molecules was kept rigid by fixing the corresponding torsion angles to their value in the crystal structures, which was regularized to the CYANA standard geometry (Gottstein et al. 2012). The rigid parts, ubiquitin (Met1–Leu71), Ubc7 (Met1–His94; Arg109–Phe165) and U7BR (Asn171–Thr224), were held together as a rigid body by employing distance restraints from the crystal structure. The remaining parts of the backbone of ubiquitin (Arg72–Gly76), Ubc7 (Ser95–Glu108), and the linker between Ubc7 and U7BR (Gly166–Glu170) as well as all the side chain torsion angles including χ^1^ could rotate freely in all calculations.

To assess how the presented conformational sampling method can create ensembles representing the provided experimental restraints, additional calculations were performed. The distance distribution of the conformation ensemble described above in comparison to a free calculation without the long-range restraints shows the expected behavior. First the sampled distance distributions without the long-range EPR derived restraints (Figure S1, blue curve) are much broader and do not show the distinct distance probabilities that were measured experimentally. Second, the distance distributions obtained from the ensembles with this new approach (Figure S1, magenta curve) are in high agreement with the experimentally measured distributions (Figure S1, green curve).

Additionally, we calculated ensembles with removing one of the experimental long–range distances from the calculation and compared the distribution of this distance in the resulting ensemble to the experimentally measured distribution (Figure S2). The conformational ensemble generated leaving out one distance constraint, reproduces the experimental distance distribution of this non-constrained distance and in most cases even the shape of the experimental distribution, showing that the created conformational ensemble reproduces the single remaining unconstrained distance and thus can model an experimental distribution that was not included.

### Visualization of the conformation space

To calculate the population distribution within the obtained conformation space, each conformation of the ensemble was weighted by a probability derived from the PELDOR distance distributions. Taking each PELDOR distance distribution as a probability density (with an integral of unity), we calculated a joint probability for any given structure. In detail, for each restraint *i* with distance *r*_*i*_ the area below the distance probability distribution in the region *r*_*i*_ *± 2.5* Å was used as the probability for the corresponding distance in the calculated structure, and for any given structure the probabilities of the PELDOR distances were multiplied to obtain an overall probability. The resulting values were afterwards divided by the maximum probability obtained in these calculations such that the structure with the highest value is assigned a relative probability of one. This yields relative probabilities that allow us to compare the relative weights of specific conformations. In order to create a representation of the structural ensemble that reflects the experimental distance distribution as a probability density function, we developed the following method. To visualize the sampled distribution of dimeric proteins the rigid parts of one of the monomers (from here on called the stationary monomer) where superimposed and aligned such that the other monomer (called the moving monomer) is positioned around the stationary monomer according to the specific conformation in the ensemble. The assigned probabilities of the moving monomer were mapped to a three-dimensional grid; for this purpose, all moving monomers in the ensemble were represented by 3D Gaussians. In case of a nearly spherically shaped protein (as e.g. for ubiquitin) it is sufficient to represent the whole protein by one single 3D Gaussian at the geometric center, whereby in order to reduce calculation time the functions can be truncated after a certain radial distance to ignore small value contributions; in the case of more complex structures, the protein could also be represented by a number of Gaussians up to one per atom. The values of the 3D Gaussians were mapped onto the respective grid points of an evenly spaced (1 Å in each direction) 3D grid, and the Gaussians for each moving monomer of the ensemble were finally merged by taking their maximum value at each grid point where they were computed.

**Fig. 3:**
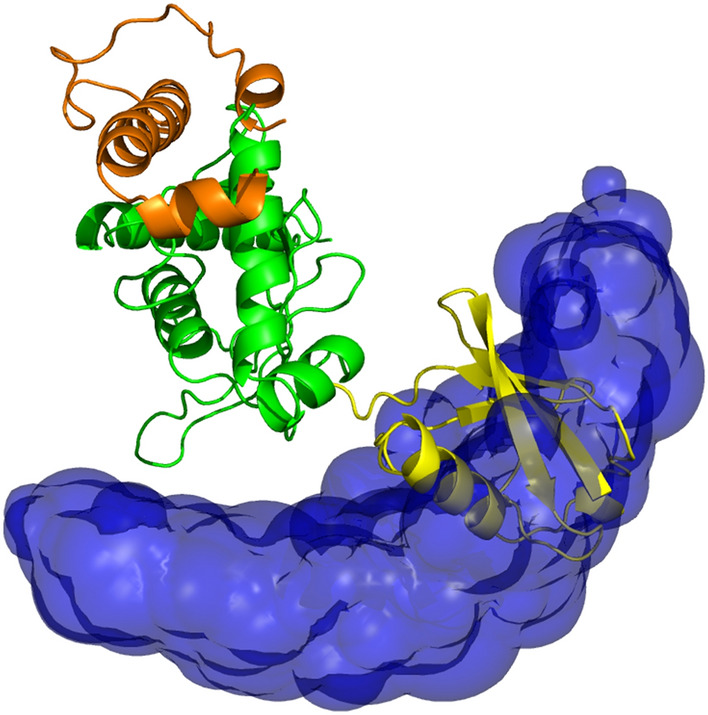
Conformation space of the Ubc7-U7BR–ubiquitin conjugate determined by PELDOR EPR spectroscopy in combination with the calculation method described here. Ubc7, shown in green and the covalently attached U7BR peptide, shown in orange, are depicted as structural models. The entire space allowed for ubiquitin according to a total of 17 PELDOR restraints measured between different sites on Ubc7-U7BR and ubiquitin is indicated as a blue-coloured volume representing Gaussians around the centre of mass of ubiquitin in the different conformations. One explicit structural model of ubiquitin in one randomly chosen orientation is shown as well in yellow. The conformation space resembles more the conformation space of the UbcH5c–ubiquitin conjugate than that of the Ubc13–ubiquitin one (Pruneda et al. 2011)

The ensemble distribution can be illustrated with PyMOL by showing contour surfaces covering regions above a threshold value for the probability grid (Fig. 3). This approach can easily be extended to a multimeric conformation ensemble, whereby all but one of the monomers are regarded as moving conformers and treated as mentioned above.

## Discussion

EPR PELDOR spectroscopy is often used to characterize large domain movements as they occur for example in membrane proteins involved in transport processes. The advantage over FRET (fluorescence resonance energy transfer) is that the EPR method does not require labelling with two different (donor and acceptor) molecules. In addition to the traditional application of PELDOR EPR for investigating the distance of macromolecular domains depending on ligand binding or posttranslational modifications, more recently EPR data have also been used to model structures of macromolecules and complexes (Duss et al. 2014; Hirst et al. 2011). Due to the requirement for the introduction of cysteines in specific locations via site-directed mutagenesis for spin labelling, the number of restraints that can be obtained from such an approach is orders of magnitude lower than restraints obtained from NMR experiments. This limitation requires the development of new software tools to model structures based on sparse restraints. Similar approaches have also been developed for the exclusive use of methyl-methyl NOEs in large proteins that cannot be completely assigned or in cross-linking coupled with proteolytic digestion and mass spectrometry methods. In the case of EPR data, the incorporation into the Rosetta software (Rosetta-EPR) has created a tool that is capable of determining the three-dimensional structure of proteins based on long-range EPR data and additional modelling (Hirst et al. 2011). An approach that combines distance and shape information available from different biophysical techniques (NMR, SAXS and EPR) was recently described (Gigli et al. 2018). The method is based on the Maximum Occurrence (MaxOcc) approach (Longinetti et al. 2006; Sgheri and Sgheri 2010) that calculates an upper bound on the statistical weight of each possible conformation. The approach was tested with paramagnetic ions (Gd^III^) bound to the two different domains of calmodulin, and it was demonstrated that PELDOR data can be used to investigate the conformation space of this protein in which two domains are linked via a flexible linker.

Here we provide another solution to the calculation of the accessible conformation space of proteins covalently linked by modifying a well-established and widely used software package for NMR-based structure determination. Our method enables the characterization of the conformation space using EPR PELDOR spectroscopy also with larger numbers of restraints. A higher number of restraints could not have been used in a previously implemented version of our CYANA-based approach due to the exponential growth of computational time with the number of PELDOR distances (Kniss et al. 2018). Here we describe an approach that circumvents this problem of exponential growth and thus provides an efficient way to calculate the conformation space based on EPR PELDOR data. While the new calculation method described here allows for the incorporation of a high number of EPR derived distances, the question remains what is the minimal number required for determination of the conformational space. This question cannot be answered for all systems as the number of restraints necessary to obtain a good representation of the entire conformation space depends on the flexibility of the system. Highly flexible ones will require more restraints than more restricted ones. A reasonable approach is to first use a relatively small number of spin pairs and calculate the conformation space. If adding an additional spin pair results in changes in the conformation space, more distances should be added.

In principle the method described here could also be combined with other restraints such as NMR or SAXS data as well as with additional modelling as is done in the Rosetta-EPR and Maximum Occurrence approaches to obtain three-dimensional structures. The approach described here, however, is not focused on determining the three dimensional structure of proteins but instead is intended for depicting the conformation space of larger systems consisting of individual, independently folded proteins that interact with each other in a transient way, such as ubiquitin moieties within different ubiquitin chains (Kniss et al. 2018) or ubiquitin bound to other proteins.

Our approach resembles other previously described enhanced sampling methods such as metadynamics. In metadynamics the sampling is biased by iteratively adding Gaussian potentials based on the system’s history, which leads to a progressive flattening of the free energy landscape (Laio et al. 2008). By interpreting the CYANA target function, which in fact is an error function, as a free energy landscape, the long–range distances from PELDOR measurements could be interpreted as collective variables of a metadynamics sampling (Laio et al. 2008). However, our method uses the long-range restraints to confine or bias the system to a specific region of the conformational space. This type of biasing has also been described in umbrella sampling (Torrie and Valleau 1977) where multiple simulations are performed with different bias potentials and the resulting biased probability distributions are then combined to obtain the unbiased free energy profile. In contrast to metadynamics, we first bias the system towards a specific region of the conformation space by additional terms of the CYANA error function and then gradually and history-dependent change this bias towards other optima.

### Supplementary Information

Below is the link to the electronic supplementary material.

Supplementary material 1 (DOCX 651.6 kb)

Supplementary material 2 (DOCX 11.6 kb)
