# Supplementary material for: Efficient determination of the accessible conformation space of multi-domain complexes based on EPR PELDOR data
Source: J Biomol NMR. 2023 Nov 15;77(5-6):261–9. doi: 10.1007/s10858-023-00426-3 (PMC10687113; doi:10.1007/s10858-023-00426-3)
Supplement: Supplementary file 1 — Supplementary material 1 (DOCX 651.6 kb) [file 10858_2023_426_MOESM1_ESM.docx]

**
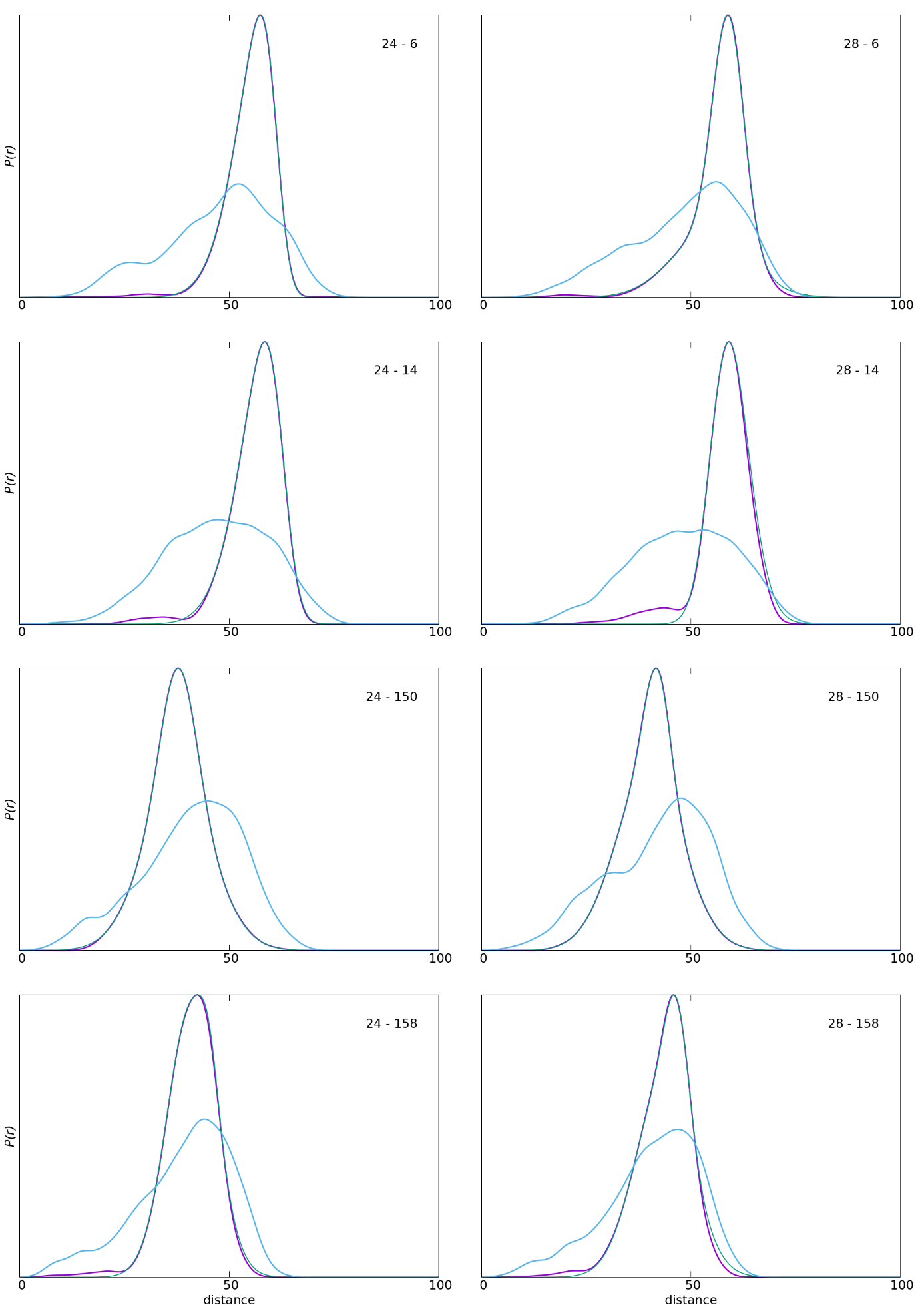
**

**Figure S1:** Distribution of a specific spin-label pair distance (indicated in the right top corner of each plot) in the calculated constraint-free ensemble (blue curve), the constrained ensemble (magenta curve), and the experimentally determined PELDOR distribution (green curve). The curves for the calculated ensembles were generated by adding up a Gaussian for each distance found in a conformation.


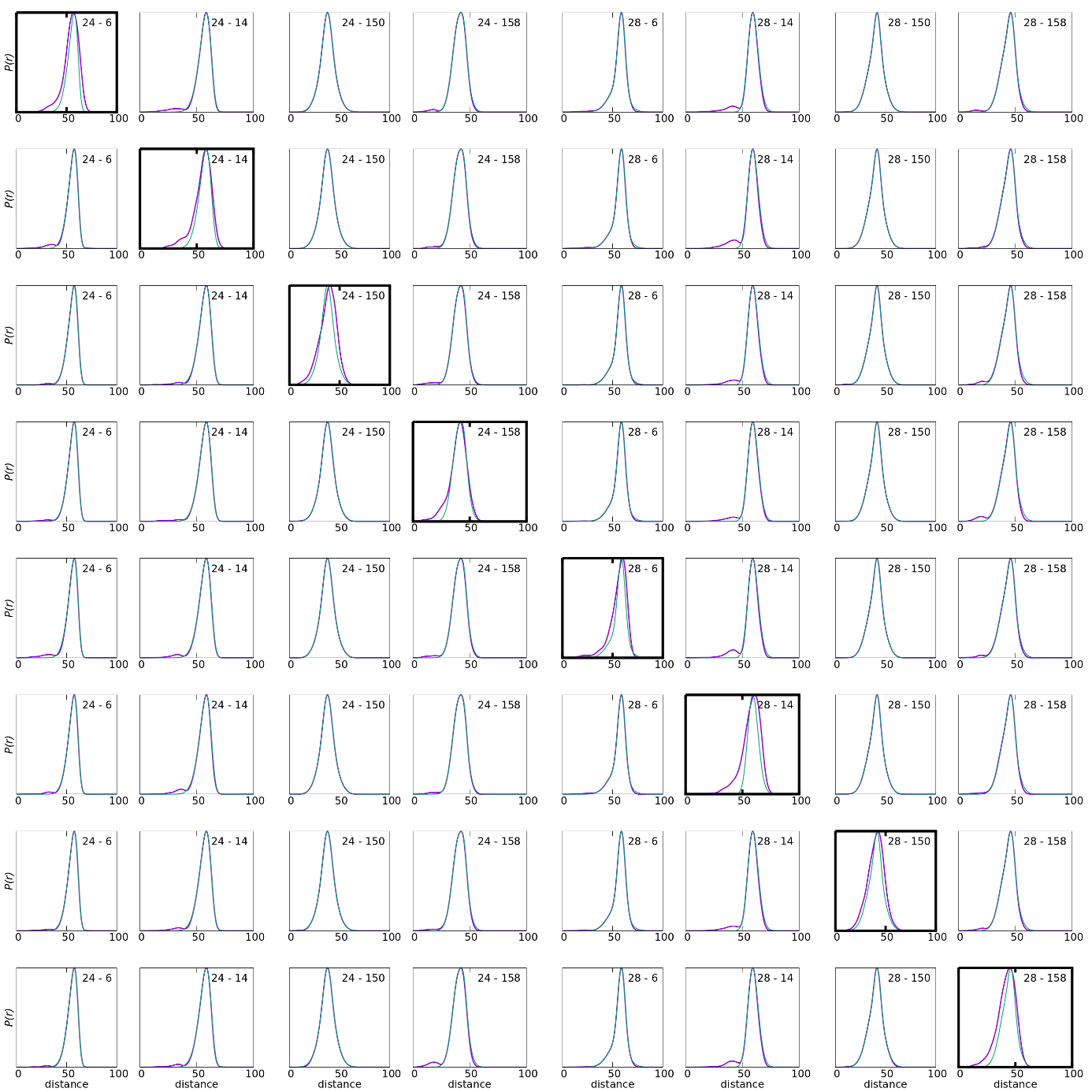


**Figure S2:** The distance distribution of the spin-label pair distances (indicated in the right top corner of each plot) for the calculated ensembles (blue curve) in comparison with the experimentally determined PELDOR distributions (green curve). Each row of plots represents the distance distribution of one respective structural ensemble when one of the experimental restraints was ignored. The distance distribution of the spin-label pair that was not restrained is marked by a thicker frame. The curves for the calculated ensembles were generated by adding up a Gaussian for each distance found in a conformation.
