# Supplementary material for: Efficient determination of the accessible conformation space of multi-domain complexes based on EPR PELDOR data
Source: J Biomol NMR. 2023 Nov 15;77(5-6):261–9. doi: 10.1007/s10858-023-00426-3 (PMC10687113; doi:10.1007/s10858-023-00426-3)
Supplement: Supplementary file 2 — Supplementary material 2 (DOCX 11.6 kb) [file 10858_2023_426_MOESM2_ESM.docx]

**Ubiquitin Ubc7U7BR**

A28C K207C

S158C

E150C

K29C

Q14C

Q6C

E24C S158C

E150C

K29C

Q14C

Q6C

E16C K207C

S158C

E150C

K29C

Q14C

Q6C
